# Direct Contact Ultrasound in Food Processing: Impact on Food Quality

**DOI:** 10.3389/fnut.2021.633070

**Published:** 2021-01-28

**Authors:** Leire Astráin-Redín, Marta Alejandre, Javier Raso, Guillermo Cebrián, Ignacio Álvarez

**Affiliations:** Departamento de Producción Animal y Ciencia de los Alimentos, Tecnología de los Alimentos, Facultad de Veterinaria, Instituto Agroalimentario de Aragón–IA2 (Universidad de Zaragoza-CITA), Zaragoza, Spain

**Keywords:** ultrasound, direct contact, quality, food compounds, nutritional compounds

## Abstract

Consumers' demand for “minimally processed” products that maintain the “fresh-like” characteristics has increased in recent years. Ultrasound (US) is a non-thermal technology that enhances mass and energy transfer processes resulting in improved food quality. A new method of applying US to food without using a liquid or gaseous medium for the propagation of acoustic waves has recently been under research. It is known as direct contact US, since the food is directly placed on a plate where the transducers are located. In this type of systems, the main effect is not cavitation but acoustic vibration, which encourages mass and energy transfer processes due to the “sponge effect.” Furthermore, as the product is not immersed in a liquid medium, the loss of hydrophilic nutritional compounds is reduced; systems such as these can thus be more easily implemented on an industrial level. Nevertheless, the very few studies that have been published about these systems mainly focus on dehydration and freezing. This article summarizes published research on the impact of direct contact US in nutritional and organoleptic quality of food in order to assess their potential to meet new market trends.

## Introduction

In recent years, consumers are demanding safe, healthy and long shelf-life products that maintain their “fresh-like” characteristics but without any chemical preservatives. However, this cannot be achieved through the application of thermal technologies, which, although longer shelf-life is possible, nutritional and quality losses are caused due to the high temperatures and long processing time. Therefore, since the twentieth century, non-thermal food processing technologies such as pulsed electric fields (PEF), high hydrostatic pressure (HPP), ultrasound (US), UV light, cold plasma and irradiation (IR) have been widely investigated (1). These technologies allow extending the shelf-life of the food but with small increase in the temperature, affecting minimally the nutritional properties, texture, color, taste and aroma of the food, which means, that products with similar characteristics to those of fresh food are obtained (2). However, despite consumers' demand for “minimally processed” products, awareness of novel technologies is still very low and there is a lack of trust in them (3).

One of these non-thermal food processing technologies is US, and its potential to improve mass and energy transfer processes has attracted great attention. Moreover, US is included within the “Green Food Processing” concept proposed by Chemat et al. (4) to refer to those technologies that allow to process food with a lower consumption of energy and water, thereby obtaining processing methods that are more sustainable and environmentally friendly.

**Figure 1:**
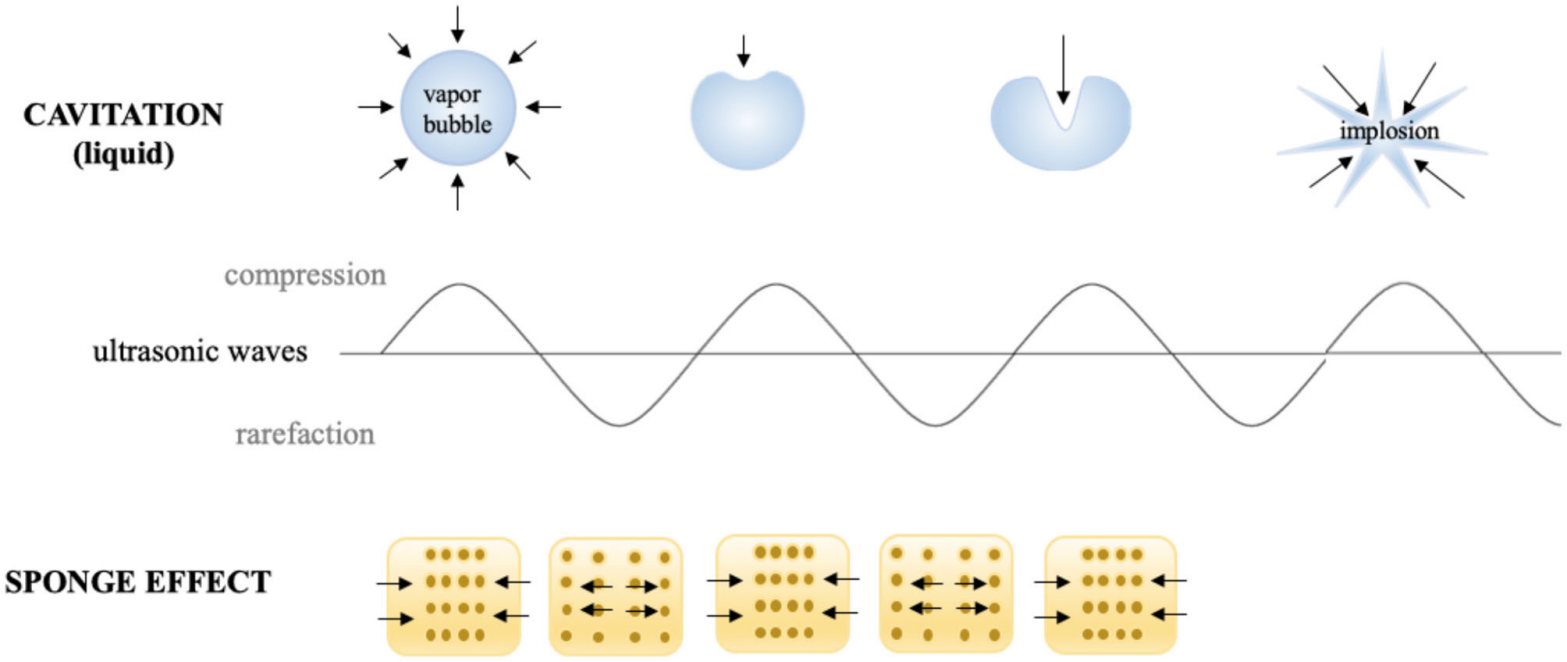
Cavitation and sponge effect due to US.

In the food industry, most research on the application of high power US (20–100 kHz, > 1 W/cm^2^) is focused on systems in which a liquid or a gaseous medium (such as air, then called airborne US) is used for the propagation of US waves (5). Most of the applications of this technology (such as cleaning, atomization, homogenization and emulsification, defoaming, drying, and freezing) are based on that manner of applying US due to its capability to produce permanent changes in the propagation medium (6). The mechanisms of action behind these effects are the cavitation phenomenon, microcurrents, microjets, the sponge effect, and the primary radicals H· and ·OH, which occur in the food matrix (Figure 1) (7, 8).

Several studies have been conducted over the last few years on the potential of ultrasound to obtain food with greater nutritional value and better organoleptic properties (9). This technology favors mass and energy transfer processes, assisting i.e., the elaboration of infusions at lower temperatures (30°C) with a higher content of total polyphenols (6–10 folds higher) and anthocyanins (8–10 folds higher) (10), and red wines with a greater content of polyphenols (11). Moreover, US also promote the elimination of compounds naturally present in food that are potentially harmful to human health, such as oligosaccharides from pulses (12) or heavy metals such cadmium from edible crabs (13), and even carcinogenic compounds such as acrylamide from fried potatoes (14). One of the most commonly used food preservation process is dehydration in which US application reduces the loss of bioactive compounds and improves the color of dehydrated products (15). In the case of freezing, in addition to reducing processing times, the US favors the formation of small ice crystals that, when thawed, reduce the loss of water, resulting in a product with better texture (16, 17).

Nonetheless, consumers not only demand “minimally processed” food, but also have great interest in functional food or nutraceutical ingredients that have additional healthy benefits beyond basic nutrition (18). However, the conventional extraction of natural food additives is quite limited due to the high-energy cost, the use of toxic solvents or the high consumption of water (19). US allows the extraction of bioactive compounds in an environmentally friendly way (4) reducing the use of solvents or with lower energetic costs. In fact, the potential of US to improve the extraction of bioactive compounds (such as polyphenols, carotenoids and anthocyanins) has been demonstrated in many studies (18–25). In addition, US also favor the extraction of functional compounds from foods that give them specific characteristics (18, 26). Within this group are the polysaccharides such as pectins (27, 28), gums (29), alginate and carrageenans (30, 31) and cellulose (32) that provide structure, stability and viscosity to the products. Finally, US also improved the extraction of proteins used to enrich food with low protein content or those used as functional additives to stabilize emulsions or foams (33–35).

Therefore, US is a non-thermal technology with great potential for the food industry and, in fact, there is already some equipment operating in industries e.g., for extraction, cutting soft products and filtration (36). However, there are still many limitations that make this not always possible, and that is why new US application systems are sought such as direct contact or contacting US system, in which the food sample is in close contact with the transducer. Differently to traditional US in which the product is immersed in a liquid, usually water, or applied to air (airborne US), US is applied in dried conditions. In this case, the acoustic vibrations that reach the solid matrix cause successive compressions and expansions of the material, which behaves as a sponge (Figure 1) (37). This mechanical stress (“sponge effect”) may result in microcracks and microchannels in the internal structure. Acoustic vibration can also improve energy transfer, as reported in different processes such as freezing, drying, etc. (38). As indicated, the main advantage of this system is that the loss of hydrophilic macro or micronutrients would be reduced (39), although this point has not been specifically investigated. In addition, it can be applied to any product without the need to be immersed in water. However, very few studies have been published in this field (40) and thus, this review focuses on describing direct contact US systems and analyzing their impact on food nutritional, quality and sensory properties.

## Direct Contact US Systems

Similar to water-immersed or airborne US systems, frequency, vibrational amplitude and power intensity are the key parameters (5) for direct contact US. In general, low frequencies are used (close to 20 kHz) where physical and mechanical effects are mostly observed. However, there is not specific studies of the effect of this parameter. Similarly, it occurs with the other parameters. Any case, associated to them, thermal effect can occur if high intensities are applied. This is a crucial parameter in direct contact US which has to be considered for its scaling-up, since it can affect product quality by losing thermosensitive nutritional compounds (i.e., ascorbic acid) or degrading certain pigments (i.e., anthocyanins, carotenoids) affecting negatively the color of the food. Although US is a non-thermal technology by definition, the US-treated product may become heated due to friction among particles, dispersion, and the viscous absorption that takes place when sound waves are transmitted through food products (41), and also due to expansions and contractions generated by the piezoelectric ceramic of the transducers (42). Due to this, to minimize the heat emitted by the transducers, a series of cooling systems or US ON/OFF activation protocols are applied (43, 44).

Several systems have been developed to apply US by direct contact in a series of different food producing processes such as drying, freezing, etc. (Table 1). All of them have the same basic elements; moreover, in all cases, the transducers or horns are in direct contact with the plate (emitter) on which the samples are placed.

**Table 1:** Different systems of application of US by direct contact with food.

**Process**	**US system**	**Study**	**Results**	**References**
Drying	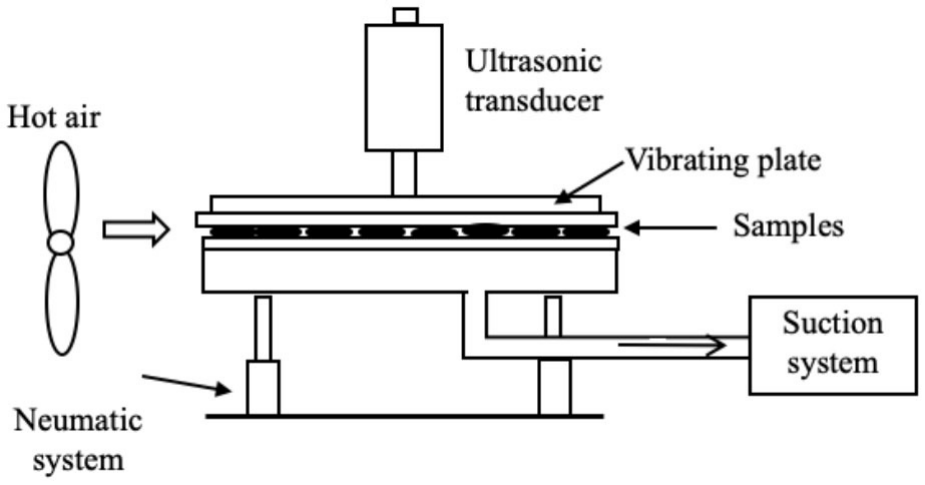 Adapted from (45)	Carrot slices US parameters: - Frequency: 20 kHz - Power: 100 W - Static pressure Conditions evaluated: - Airflow: 1 and 3 m/s - Air temperature: 22°C	Improvement in the drying rate (70.0%) Lower final moisture	(45)
Drying	The same as (45) 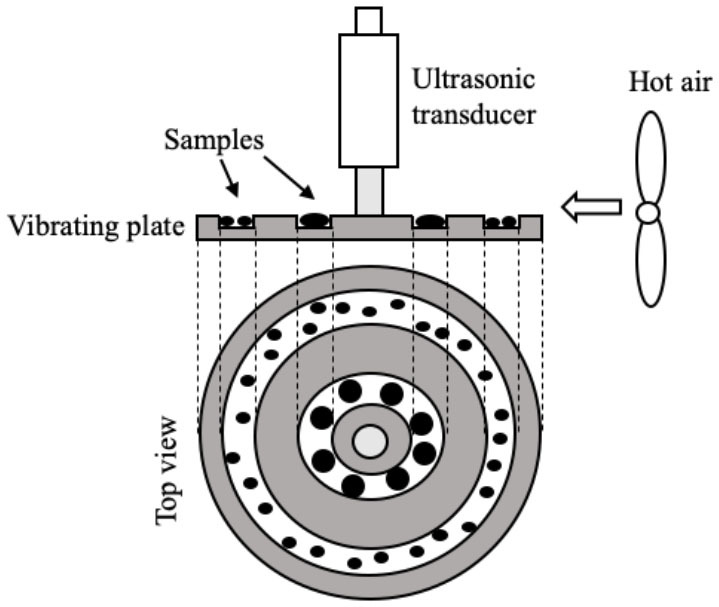 Adapted from (46)	Carrot, apple, and mushroom slices US parameters: - Frequency: 20 kHz - Power: 100 W - Pressure static (1): 0.05 kg/cm^2^ Conditions evaluated: - Airflow: 1.7–2 m/s - Air temperature: 20 and 55°C	Reduction of drying time (carrots: up to three times, apples: 50.0–76.7% and mushrooms: 68.3–83.3%) Reduction of drying time (carrots: 50.0–58.3%, apples: 66.7–233.7% and mushrooms: 50.0–75.0%)	(46)
Drying	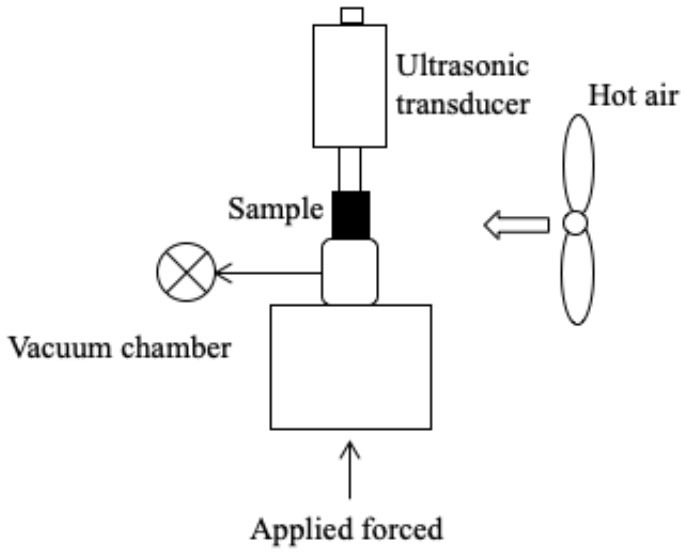 Adapted from (47)	Apples and potatoes slices US parameters: - Frequency: 20 kHz - Power: 25 and 50, W - Static pressure: 0.0155–0.050 kg/cm^2^ - Suction pressure: 10 and 20 mbar Conditions evaluated: - Airflow: 1 m/s - Air temperature: 31°C	Increase in the effective diffusivity coefficient	(6, 37)
Drying	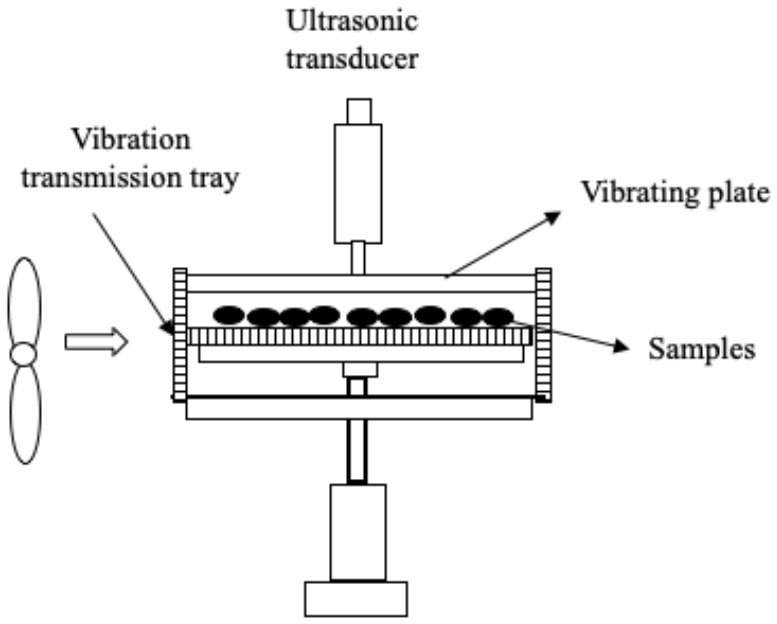 Adapted from (48)	Apple slices US parameters: - Frequency: 20 kHz - Power: 75 and 90 W Conditions evaluated: - Air temperature: 40 and 60°C - RH% air: 25% - Airflow: 1 m	Reduction of drying time (46.0–57.0 %) No differences in texture	(48)
Drying	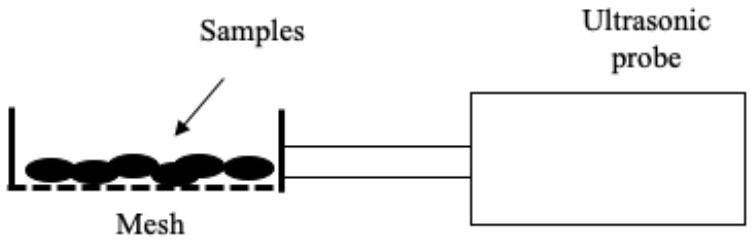 Adapted from (43)	Red bell peppers and apples US parameters: - Frequency: 24 kHz - Power: 42 W - Effective amplitude: 6–13 μm Conditions evaluated: - Air temperature: 70°C - Continuous US treatment - Intermittent US treatment: • 50% net sonication time • 10% net sonication time	No impact on final relative water content Intermittent US treatment at net sonication of 10 % did not improve the process, but at net sonication of 50% there was a reduction in drying time (18–20%) Continuous US treatment allowed to reduce drying time (18–27%)	(43)
Drying	The same as (43)	Potato cylinders US parameters: - Frequency: 24 kHz Conditions evaluated: - Air temperature: 70°C	US effect was strongest in the outermost layer (0.0–0.6 mm) and at the sonicated surface US treatment allowed to reduce drying time (by 10.3%)	(49)
Drying	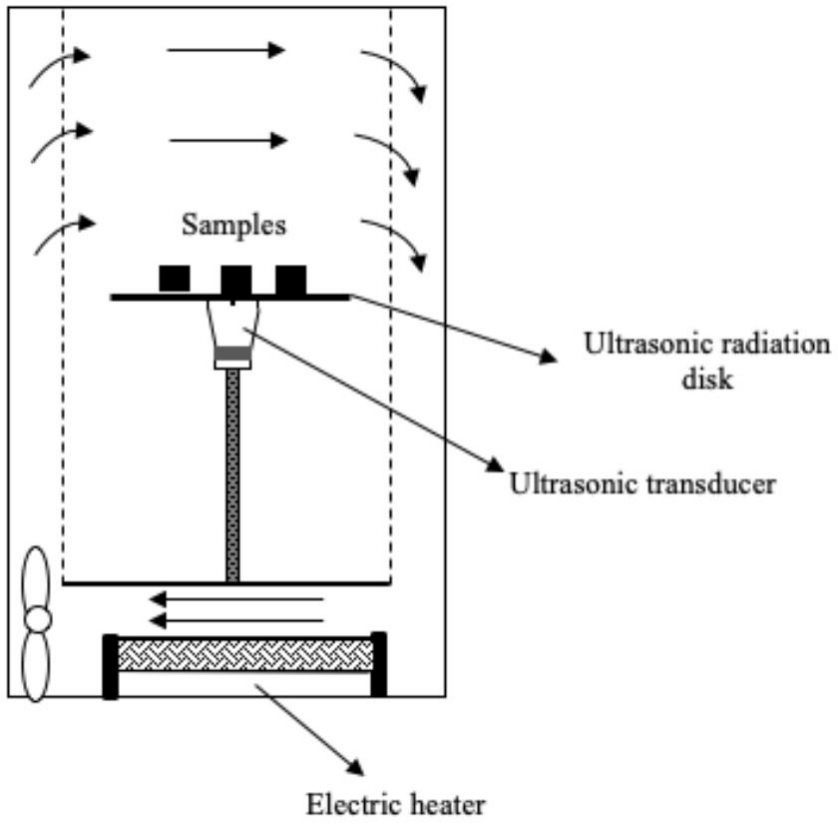 Adapted from (50)	Purple-fleshed sweet potato slices US parameters: - Frequency: 28 kHz - Power: 30 and 60 W Conditions evaluated: - Air temperature: 40, 50, 60, and 70°C - Airflow: 1 m/s	Drying time was reduced by increasing the US power (31.5–47.7 %) but the US effect was less pronounced at higher air temperature The drying rate was improved (50.8–100.0 %) at high US power and low temperature Increase in the effective moisture diffusivity (D_eff_) (17.6–48.1%) Distortions of the cellular tissue and the appearance of large cavities Improvement of the rehydration capacity	(50)
Drying	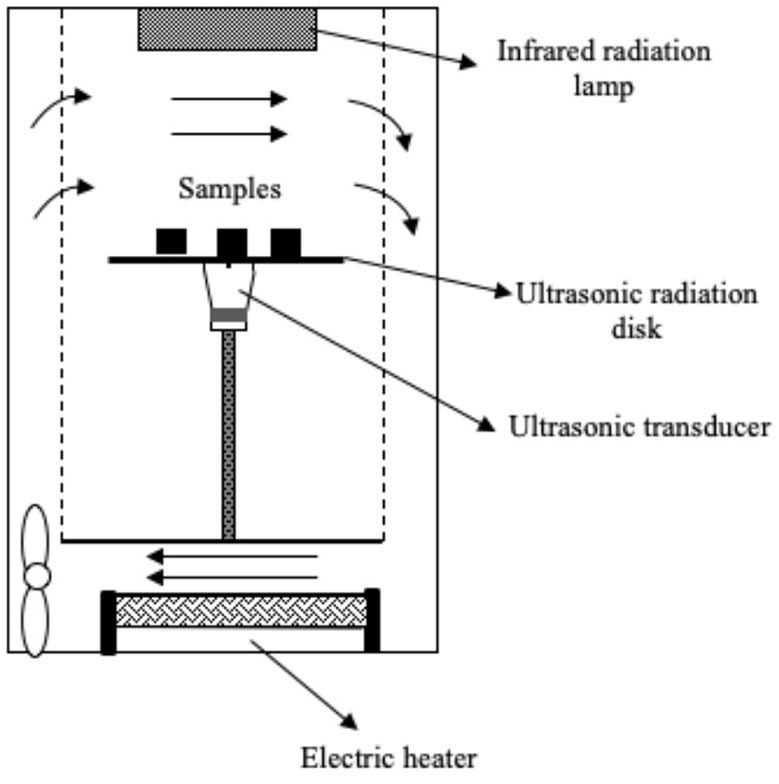 Adapted from (51)	Pear slices US parameters: - Frequency: 28 kHz - Power: 30 and 60 W Conditions evaluated: - FIR power: 100, 220, and 340 W - Air flow: 1.5 m/s	Increase in the drying rate (at 45°C, the increase was 33.3% at 24 W and 140.1% at 48 W) Positive impact on total phenolic content, flavonoids, and ascorbic acid Appearance of more numerous and larger microchannels in the cell tissue	(51)
Drying	The same as (51)	Kiwi slices US parameters: - Frequency: 28 kHz - Power: 18, 36, and 54 W Conditions evaluated: - FIR temperature: 120, 200, and 280°C - Airflow: 1.5 m/s	Reduction of drying time (the increase at 120, 200, and 280°C was 32.2–48.4%, 22.2–38.9%, 14.3–33.3%, respectively) The drying rate was improved (66.7%) by increasing US power US decreased the resistance to internal diffusion, facilitated the migration and removal of the immobilized and bound water	(52)
Drying	The same as (50)	Pear slices US parameters: - Frequency: 28 kHz - Power: 24 and 48 W Conditions evaluated: - Air temperature: 35, 45, and 55°C - Airflow: 1 m/s	Best increase in drying rate (33.3–140.1 %) at low air temperature The microstructure of the pear samples showed more numerous and larger cavities Positive impact on total phenolic content, flavonoids, and vitamin C Improvement in rehydration capacity	(53)
Drying	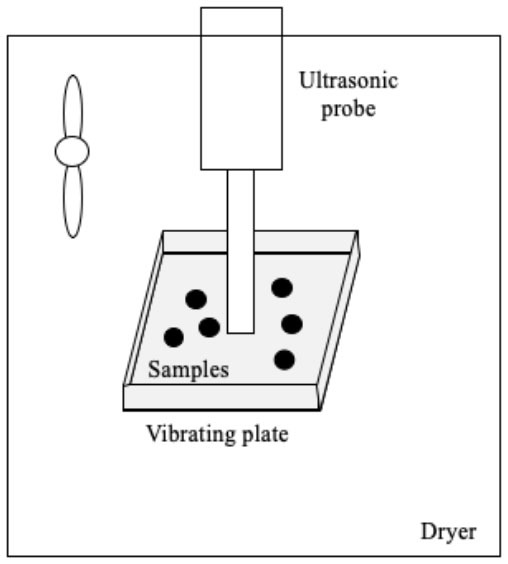 Adapted from (54)	Garlic (*Allium sativum* L.) US parameters: - Frequency: 20 kHz - US treatment: 3 s on/ 1 s off Conditions evaluated: - Power: 216.8, 902.7, and 1513.5 W/m^2.^ - Air temperatura 50, 60, and 70°C - Airflow: 2.5 m/s	Reduction of drying time (the increase at 216.8, 902.7, and 1513.5 W/m^2^ was 5.0%, 12.5%, 35.0% respectively, at 50°C) The drying time was reduced by increasing air temperature Positive impact on thiosulfinate and TPC at 216.8 and 902.7 W/m^2^ Greater retention of organosulfur compounds Color improvement	(54)
Drying	The same as (54)	White cabbage (*Brassica oleracea* L. variety Capitana L.) US parameters: - Frequency: 20 kHz - US treatment: 4 s on/2 s off Conditions evaluated: - Power: 492.3 and 1131.1 W/m^2^ - Air temperature: 60 °C - Airflow: 2.5 m/s - Pre-blanching treatment (100°C/30 s)	Synergistic effect of blanching and subsequent US drying to intensify drying process No color differences Higher TPC (12.6 %) in un-blanched sonicated samples at 492.3 W/m^2^ No positive effect on Vitamin C content No clear effect on glucosinolate	(55)
Freezing	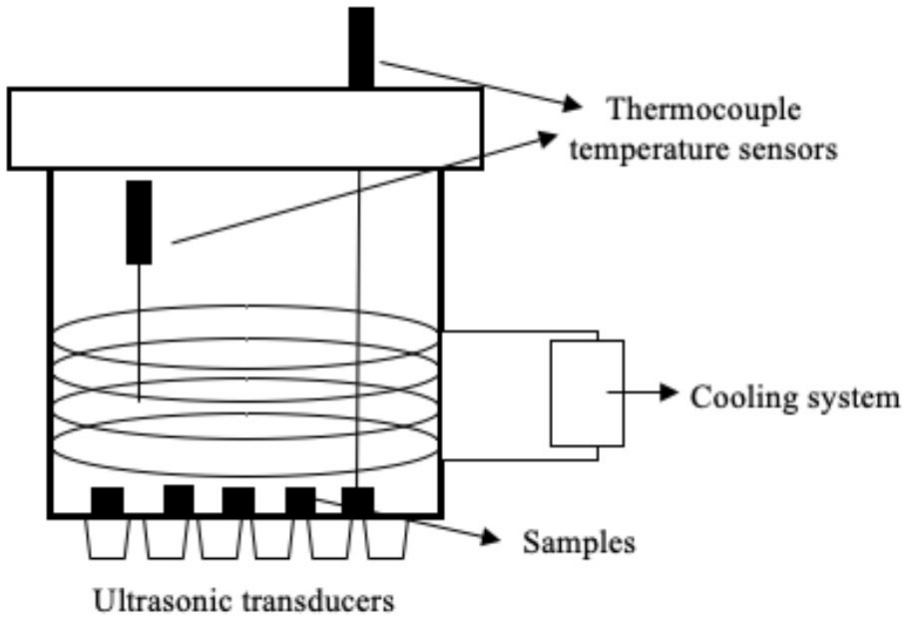 Adapted from (56)	Mushroom (*Agaricus bisporus*) US parameters: - Frequency: 20 kHz - Power: 300 W - 12 transducers Conditions evaluated: - US treatment: 10 s on/20 s off when the sample temperature reached −1°C - US treatment: 10 s on/ 10 min off during 3 weeks of frozen storage	Earlier nucleation Smaller crystal size and more uniform shape The microstructure was more uniform, featuring more numerous and more dense pores	(56)
Freezing	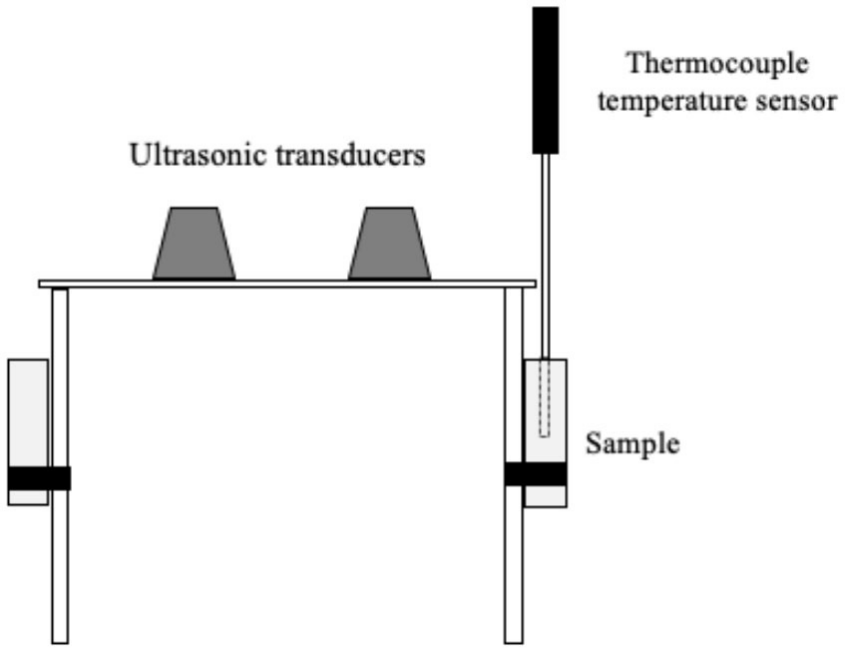 Adapted from (57)	Chicken breasts US parameters: - Frequency: 40 kHz - Power: 50 W Conditions evaluated: - US treatment: 3s on/ 5s off throughout the entire freezing process - Air temperature −13 to −25° C - Air flow: <0.4 m/s	Reduction of freezing time (19.9%) No difference in quality attributes such as WHC, CL and protein digestibility	(57)
Freeze-drying	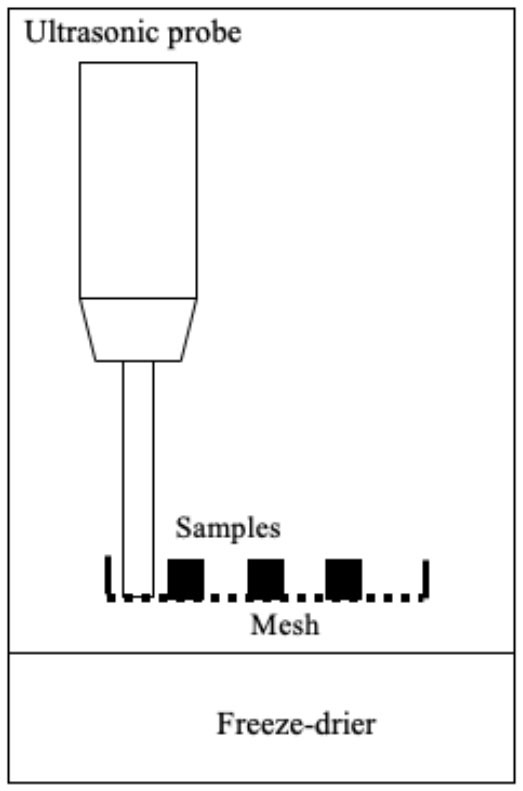 Adapted from (58)	Red bell peppers Samples were frozen in a cooling chamber to reach a temperature of −20°C Then they were dried by applying US US parameters: - Frequency: 20 kHz Freeze-drying pressure was 46 Pa Conditions evaluated: - Power: 76, 90, and 110 W - Net sonicated time: continuo (100%), 25%, 14% and 10%	Minimum US thermal effect at 76 W and net sonication time of 10 % Reduction of drying time No difference in quality attributes such as bulk density, color, ascorbic acid, and rehydration capacity	(58)

### Drying

Drying is a preservation process that has a great effect on organoleptic properties and heat-sensitive nutritional compounds such as antioxidants and vitamins (39, 59). Numerous studies have focused on the study of US-assisted drying of fruits and vegetables and its effect on the physical (water activity, shrinkage, rehydration, color, porosity, among others) and chemical (nutrients, antioxidants, vitamins) quality of the dried product (60–62).

All the studies included in Table 1 reported that US improved the drying rate (up to 70% in some cases), reduced drying time, and enhanced the quality of the dehydrated food. For example, Liu et al. (50) studied the impact of contact-US-assisted drying (28 kHz) on the color of purple-fleshed sweet potato slices by applying 30 W and 60 W US treatments and four air temperatures (40, 50, 60 and 70°C). The effect of the US was more noticeable at high temperatures, as drying times were greatly reduced: at 70°C, time reduction was 18.7 % (30 W) and 37.5 % (60 W), and the dried potato samples were brighter, redder, and less yellowish than control. Tao et al. (54) also observed improvements in the color (whiter values) of dried garlic assisted by US reducing drying time by 48.5% at 60°C. Similar conclusions have been reported using airborne systems for carrots (63), strawberries (64), and green peppers (65).

An important aspect to be considered in the traditional heat-dried process is the potential loss of thermosensitive bioactive compounds (66). Thus, the reduction of drying times by accelerating mass and energy transfer processes minimizes the loss of nutritional compounds. Liu et al. (51, 52) studied the effect of direct-contact-US-assisted convective drying on total phenolic content (TPC), flavonoids, and ascorbic acid of pear slices by applying hot air flow (35, 45 and 55°C) or far infrared radiation (FIR) (100, 220, 340 W). It was observed that the higher the ultrasonic power the lower the loss in TPC: e.g., at 45°C and ultrasonic powers of 24 and 48 W, the retention of TPC was 14.7 and 39.7%, respectively, whereas at 220 W FIR and ultrasonic powers of 30 and 60 W, the improvement compared to control was 6.7 and 16.7%, respectively. However, no beneficial effect of US was observed at 55°C and 340 W FIR; it even had a negative effect as compared to control. According to the authors, this was related to oxidation reactions, since at elevated temperatures the tissue was more sensitive to damage; when US was applied, associated mechanical effects could intensify heat damage, while oxidative reactions occurred more easily due to increased contact between phenolic compounds and oxygen (67). Similar results were found for flavonoids when US was applied at low temperatures (35°C; 48 W US) or at low FIR powers (100 W, 220 W; 60 W US), thereby leading to increases in flavonoid content of 21.1, 45.5 and 26.6%, respectively. However, at higher temperatures or FIR powers, the effect of US was harmful. The effect of the US treatment on ascorbid acid content was always positive, and increased along with power. The highest ascorbic acid contents were observed at 35°C and 48 W US (US samples, 42.5 mg vitamin C/100 g vs. non-US samples, 30.0 mg vitamin C/100 g), and at 100 W FIR and 60 W US (US samples, 265.5 mg ascorbic acid/100g vs. non-US samples, 226.1 mg ascorbic acid/100 g). Another example is that of Tao et al. (54) applying 20 kHz-US during the drying at 60°C of garlic slices (*Allium sativum* L.). Garlic has healthy benefits associated with thiosulfinates that have anti-inflammatory, antioxidant and antimicrobial properties (68). In this study, the total thiosulfine content was 16% higher at an ultrasonic intensity of 902.7 W/m^2^ compared to non-sonicated samples, The TPC was also improved at 902.7 W/m^2^ (12 %), while at a higher ultrasonic intensity (1,513 W/m^2^) the content was even lower than control. Nevertheless, the antioxidant capacity was very similar between non-sonicated and sonicated samples, showing a small improvement when applying 902.7 W/m^2^. The application of direct contact US to food drying systems can therefore increase the retention of thermosensitive bioactive compounds but the treatment conditions need to be optimized, mainly the ultrasonic power.

Finally, in the studies by Liu et al. (50, 52) it was observed that rehydration capacity, one of the most important parameters that defines the quality of dehydrated food (69, 70), was improved by 10.6 % in samples of purple-fleshed sweet potato dried at 40°C and 60W (US), and by 36.4, 15.7 and 13.2% in samples of pear slices dried at 35, 45 and 55°C, respectively, and applying a US power of 48 W. These results can be explained by the fact that the application of US by direct contact in solid food, as reported in liquid immersion systems and air systems (19, 71), leads to the formation of cavities and microchannels in plant tissues via mechanical effects (49–52) that reduce internal resistance to the flow of water and enhance its incorporation during rehydration.

### Freeze-Drying

Freeze-drying is a process widely used to obtain high-quality dehydrated food by preserving shape and color while minimizing the loss of nutrients (72). However, extended processing times and high energy costs are involved. The application of US could thus serve as a useful alternative in order to accelerate mass and energy transfer process. To the best of our knowledge, only one published study deals with the application of US by direct contact to vacuum freeze-drying. This would probably be due to the technical difficulties involved in applying US in vacuum freeze-drying systems (58). US equipment in that study consisted in two sonotrodes, in the tip of which a mesh was fixed to hold the samples (Table 1). An intermittent (10s on/90s off) application of US (from 76 to 110 W) led to a reduction in the freeze-drying time of red bell peppers of 11.5%, but no differences were observed in terms of rehydration capacity, bulk density, color, or ascorbic acid content of the treated samples compared to the conventionally freeze-dried samples. Since the application of US in the freeze-drying process allows reducing the processing time, it is necessary to conduct more studies to evaluate its impact on the nutritional and organoleptic quality of the food.

On the other hand, it is worth mentioning that US airborne systems have been tested in atmospheric drying at low temperature processes (an alternative to freeze-drying) with the aim of improving the quality of air-dried food. Bantle and Eikevik (44) did not observe any differences in color or shrinkage of green peas when US was applied. Similarly, Colucci et al. (73) investigated the impact of US-assisted atmospheric drying at freezing temperatures on the antioxidant properties of eggplant samples, and likewise did not find significant differences when applying US (25 and 50 W). Moreover, although differences were not significant, the application of US promoted the degradation of ascorbic acid (1.5–7%), TPC (4.2–15%) and antioxidant capacity (3–13.8%) in samples dried at −10°C and 2 m/s.

### Freezing

The quality of frozen food is determined by the shape, location, and distribution of ice crystals inside the product (74, 75). Therefore, rapid freezing is sought in order to allow the formation of small and numerous intra- and extra-cellularly located ice crystals that minimize quality losses after thawing (76, 77). Many studies have shown that immersion freezing in ultrasonic baths improves the quality (microstructure, weight loss, texture, color, and nutritional components) of frozen food by promoting the initiation of nucleation, thereby controlling the growth of ice crystals and accelerating the transfer of mass and heat (19, 40, 78).

Islam et al. (56) studied the effect of direct contact US on the freezing process of mushroom (*Agaricus bisporus*) cubes by applying a frequency of 20 kHz and a power of 300 W with intermittent treatment of 1 s on/20 s off once the sample temperature reached −1°C, and treatments of 10 s on/10 min off in the course of storage during 3 weeks. They observed that the sonicated samples displayed earlier nucleation at temperatures of −2.0 ± 0.05°C compared to control samples, in which it occurred at −2.6 ± 0.01°C. Differences in morphology and size of the ice crystals were also detected by cryo-electron microscopy. The crystals of the sonicated samples were smaller, thinner, and columnar shaped, while those of control were larger, more irregular, and featured dendrites. Although no quality parameters were analyzed in this study, the characteristics of the ice crystals strongly suggest that the US-assisted process would have a lower impact on the quality of the frozen/thawed products. Recently, Astráin-Redín et al. (57) studied the influence on Water Holding Capacity (WHC), Cook Loss (CL) and protein digestibility of meat when applying direct contact US (40 kHz, 50 W) in freezing chicken breasts while applying an intermittent US treatment. No differences in terms of those quality parameters were observed between sonicated and control samples. These results may be due to the fact that the sample size was small (5–6 g), and, although US-assisted freezing was more rapid (9.9–11.3%), the process was already rapid enough in the control samples to have a negative impact on quality. Indeed, in larger pork loin samples (120 g), Zhang et al. (79) observed smaller and more uniformly distributed crystals resulting from immersion US freezing (180 W and 30 kHz), and they obtained 61% and 12.3% lower weight losses after thawing and cooking, respectively, when applying US compared to a forced air system. Moreover, Li et al. (17) evaluated the influence of immersion-US-assisted freezing (20 kHz) on chicken breast meat, and observed an increase in the proportion of water retained within the myofibrillar protein, thereby resulting in a higher WHC.

## Conclusions

This review summarized the current state of knowledge regarding a new method of applying US to food samples, known as direct contact US systems. Although very few articles have been published on this subject, the application of US has already achieved considerable improvements in mass and energy transfer processes in the food industry, such as dehydration and freezing. In the case of dehydration, the application of US leads to a reduction in drying times, resulting in dehydrated food with a higher content of TPC, flavonoids and ascorbic acid, as well as improved sensory attributes such as color, along with improved functional properties (i.e., rehydration). However, most of the studies did not analyse the thermal effect that these systems could have on the samples; thus, the effect of the US treatment cannot be evaluated correctly as it can be hidden or misleading. As far as freezing processes are concerned, it has been reported that direct US contact freezing promotes the formation of small ice crystals, although no improvements in certain highly relevant quality parameters of defrosted foods such as WHC and CL have been observed. For all these reasons, the application of US by direct contact can be regarded as a thoroughly useful technique to improve mass and energy transfer processes of food. However, due to the scarce number of articles on this subject, further research is required in order to gain a better understanding of this system's effect on food nutritional and organoleptic quality.

## Author Contributions

LA-R: conceptualization and writing—the original draft. MA and JR: writing—review and editing. GC: writing—review and editing and supervision. IÁ: conceptualization, writing—review and editing, and supervision. All authors contributed to the article and approved the submitted version.

## Conflict of Interest

The authors declare that the research was conducted in the absence of any commercial or financial relationships that could be construed as a potential conflict of interest. The handling editor declared a past collaboration with the authors IÁ and JR.
